# Thromboembolism in Malignant Musculoskeletal Tumour: A Literature Review

**DOI:** 10.1155/2021/6678167

**Published:** 2021-02-19

**Authors:** Achmad Fauzi Kamal, Didi Saputra Ramang, Marcel Prasetyo

**Affiliations:** ^1^Department of Orthopaedic and Traumatology, Faculty of Medicine Universitas Indonesia, Cipto Mangunkusumo General Hospital, Jakarta, Indonesia; ^2^Department of Radiology, Faculty of Medicine Universitas Indonesia, Cipto Mangunkusumo General Hospital, Jakarta, Indonesia

## Abstract

Malignant musculoskeletal tumour may cause considerable burden to general health. The fast growth combined with the tumour characteristics and its invasion capability resulted in the poor prognosis of malignant musculoskeletal tumour. Malignant musculoskeletal tumour may cause significant disability by destroying normal tissue that plays important role in body kinematics. Thromboembolism, including deep vein thrombosis, pulmonary embolism, and other kinds of venous thromboembolism, is one of the most underestimated complications of musculoskeletal tumour. Normally, thrombosis ensues when pathologic factors overcame the body hemostatic regulatory capabilities, which will predispose the body to the formation of thrombus. Venous thromboembolism in musculoskeletal tumour may develop as a result of interaction between the tumour pathologic capabilities and its interaction with normal bodily functions. In this study, we reviewed the burden of musculoskeletal tumour and its complication on global health. Then, the review will focus on the pathologic and clinical aspect of thromboembolism in malignant musculoskeletal tumour, including pathophysiology, diagnosis, and treatment based on recent findings and literature.

## 1. Introduction

In recent years, malignant cancers are currently on the rise with an estimated incidence of 15.2 million of new cases of cancer with a death count of 8.8 million for year 2015. In terms of incidence, musculoskeletal tumours are relatively rare compared to other types of cancer in adults and are more often seen in pediatric and adolescents. Musculoskeletal tumour only makes up for around 0.2–0.5% of all tumour cases in adults, and the distribution varies greatly across the globe. Musculoskeletal tumour can be differentiated into benign and malignant tumours based on the tumour invasiveness, rate of growth, and many other different factors. Compared to its benign counterpart, malignant musculoskeletal tumour generally grows much more extensively in a faster rate. If not adequately treated as soon as possible, malignant musculoskeletal tumour mainly has fatal outcome. Osteosarcoma and Ewing sarcoma are two of the most encountered types of musculoskeletal tumour, and both types are malignant. The mainstay treatment for malignant musculoskeletal tumour is complete excision of the tumour followed by reconstruction of tumour-associated defects. Limb-salvaging treatment options are much more preferable than ablative treatments [1].

Some complications are associated with malignant musculoskeletal tumour, ranging from surgical site infection after tumour excision to loss of limb function and even mortality. One of the most concerning complication associated with malignant musculoskeletal tumour is thromboembolism, including pulmonary embolism and deep vein thrombosis. Thromboembolism is deeply correlated with both orthopedic surgery and orthopedic tumour. Literature studies reported the incidence of thromboembolism in orthopedic cases ranging from <1 to 22% [2]. Symptomatic thromboembolism is associated with poor prognosis, and Iwata et al. found the incidence rate of symptomatic thromboembolism to be 1.18% in patients with bone and soft tissue sarcomas [3]. Morbidity and mortality caused by thromboembolism urged the need for a rapid and accurate diagnostic method for diagnosing thromboembolism in malignant musculoskeletal cancer patients. At the same time, it also signals the importance of early treatment of thromboembolism in malignant musculoskeletal tumour in order to avoid unwanted disabilities.

## 2. Methods

Comprehensive literature search was done by searching PubMed, Scopus, EBSCOHost, ProQuest, ScienceDirect, SpringerLink, and Wiley on November 2020. The literature search included all articles regarding thromboembolism in malignant musculoskeletal tumour, including systematic reviews, clinical trials, cohorts, case controls, and case reports. Articles on different aspects of thromboembolism in malignant musculoskeletal tumour including pathophysiology, diagnosis, and treatment modalities are included in this study in order to provide comprehensive review about thromboembolism in malignant musculoskeletal tumours. Letters, editor opinion, and commentary studies are excluded. Analysis of the articles obtained from the search is done on December 2020. Findings are screened and extracted by a single reviewer. The result of the literature search is described and summarized in Table 1.

## 3. Literature Search Results

### 3.1. The  Mechanism of Thromboembolic Formation

The exact mechanism triggering clotting in patients with thromboembolism is not fully understood. It is thought that arterial thrombus may be incited by disruption of endothelial integrity, while venous thromboembolism was found to be preceded by venous stasis. It is agreed that intact vasculature microstructure is vital in preventing thromboembolism, and disturbance of the vessel wall integrity may initiate pathophysiologic cascade that resulted in the formation of thrombus, which may get unattached to form emboli that travel and block vessels smaller in size compared to the emboli distant to the thrombus formation location. Hemostasis is the balancing process that maintains the blood flow system inside blood vessels. When pathologic factors exceeded the protective and regulatory features of hemostasis, blood coagulation will be initiated. Blood coagulation is a very complex process that happens as a result of interplay between many different intravascular elements, including shear stress on the inner lining of the vessel wall, blood coagulation factors, and other contributing agents, which ends in the generation of thrombus. Physiologically, thrombosis is actually an important protective sequence that protects the body from excessive blood loss [4, 5].

In general, thromboembolism may happen as a combination of dysfunctional vascular endothelium, activity of an intravascular tissue factor, the existence of anticoagulant endovascular proteins, and the inhibition of fibrinolysis. Normally, endothelium prevents the formation of thrombus by producing thromboregulator agents, including nitric oxide, prostacyclin, and ectonucleotidase CD39. Following endothelial disruption, exposure of collagen and intravascular tissue factor to blood will initiate the generation of thrombus by accumulation and activation of accumulated thrombocytes. Collagen and intravascular tissue participated in the thrombus regulatory equilibrium by acting as hemostatic barrier in order to maintain the integrity of the circulatory system. Intravascular tissue factor, a transmembrane glycoprotein, is expressed in vessel wall tissue and functions as a receptor and cofactor of factor VII that ultimately plays vital role in the activation of factor IX [6]. Intravascular tissue factor may also activate platelets in the absence of endothelium disruption by cleaving protease-activated receptor-4 on the surface of platelets, thereby causing platelets to release thromboxane A_2_, adenosine diphosphate, and serotonin that will activate other platelets and enhance thrombus formation [7]. Anticoagulant endovascular proteins including thrombin-activatable fibrinolysis inhibitor (TAFI), antithrombin, protein S, and protein C are a group of proteins that hinder thrombosis by inactivating blood-clotting factors including factor VIIIa and factor Va. Endogenous fibrinolysis is another protective factor that broke down formed thrombus by the activation of plasminogen into plasmin. Plasmin degrades the fibrin clot into soluble products and plays a major role in fibrin removal [8, 9].

### 3.2. Thromboembolism in Musculoskeletal Tumours: Pathophysiology

The risk of venous thromboembolism in cancer patients is almost five-fold higher compared to general population [10]. In cancer patients, it is thought that increased risk of thrombosis is attributed to some factors coined as Virchow's triad, including stasis of the blood flow, endothelial dysfunction, and state of hypercoagulability. Malignant musculoskeletal tumour patients may have limited mobility resulting from the invasion and the growth of tumour tissue. The immobilization of musculoskeletal tumour patients may cause stasis in the blood flow, contributing to the formation of thrombus. Other factors that contribute to the immobility of musculoskeletal tumour patients include the disability of the patients in the advanced stage of the disease and the damage to the musculoskeletal tissue caused by surgery. Decreased blood flow participated in the formation of blood clot by causing improper clearance of blood-clotting factor while also increasing blood viscosity. The accumulation of blood clotting factors combined with the slow blood flow may overwhelm anticoagulant regulatory systems, resulting in the hypercoagulable state of musculoskeletal tumour patients and therefore predisposing musculoskeletal tumour patients to the formation of thromboemboli. The invasion of tumour into the vascular tissue or treatment interventions for the tumour such as vascular catheters and surgery might also disturb the endothelial integrity, favoring the formation of thromboemboli [4, 11].

Some literature studies found that the musculoskeletal tumour itself may contribute to the formation of thrombus. Tumour tissue can induce hypercoagulable state by increasing the activation of coagulative factors and inactivating the anticoagulant factors at the same time. Musculoskeletal tumour also hampers the fibrinolytic cascade by upregulating plasminogen activator inhibitor-1 (PAI-1), producing proinflammatory cytokines including IL-6 and TGF-*β* which were found to induce the hypercoagulable state by increasing the expression of intravascular tissue factor, and increases platelet aggregation on the vessel [12]. Modalities used in anticancer therapies such as central venous line, blood transfusion, chemotherapy, and surgical therapy which were also found to increase the risk of thrombus formation [13]. Yoshimura et al. found that the risk of venous thromboembolism postoperatively in patients receiving surgery for malignant bone and soft tissue tumours in the lower extremity is increased, with high body mass index (BMI) which was found to be a significant risk factor contributing to the formation of thrombus [14]. Groot et al. found that the risk of postoperative venous thromboembolism increased significantly in patients receiving surgery for the treatment of long bone metastases [15].

Thrombosis may be one of the first signs of undetected neoplasm. The likelihood of the existence of tumour in patients with bilateral deep vein thrombosis is almost five times higher than in general population. Patients with metastatic cancers were especially predisposed for venous thromboembolism even when compared to other cancer patients without metastases. The Vienna Cancer and Thrombosis Study found an increased cumulative probability of venous thromboembolism in metastasized cancer patients [16, 17].

### 3.3. Diagnosing Thromboembolism in Musculoskeletal Cancer Patients

Thrombus in cancer patients is mainly diagnosed by imaging modalities. Imaging modalities that can be used include CT, MRI, Doppler ultrasound, and positron emission tomography. Contrast-enhanced MRI may be used to find intraluminal soft tissue enhancement along the suspected vessel [18]. Ultrasound imaging may be used to diagnose the tumour initially and followed up using CT or PET [19]. Laboratory diagnostic for measuring coagulation markers such as D-dimer was found to be of no use in diagnosing thromboembolism in cancer patients. Yoshimura et al. found that only 1 case out of 8 VTE cases diagnosed using enhanced CT was found to have elevated level of D-dimer above 10 *μ*g/mL [14]. However, Kaiser et al. found that high preoperative white blood cell count is a significant independent risk factor for venous thromboembolism in primary osteosarcoma patients. This signals the utility of routine preoperative blood examination in musculoskeletal tumour patients to help determine which patients may develop venous thromboembolism after surgical intervention [20].

### 3.4. Treatment of Thromboembolism in Musculoskeletal Tumour Patients

Vascular surgery is one of the main treatments for thromboembolism in musculoskeletal tumour patients. Venous thrombectomy is one of the most used procedures, where the intravascular thrombus is taken out from the blood vessel. However, recent advances in medical science noted the availability of pharmacological agent as treatment and prevention modality for thromboembolism in cancer. Haase et al. found that the usage of tranexamic acid in cancer patients undergoing endoprosthetic reconstruction decreased the number of patients with venous thromboembolism postoperatively. However, the result was statistically insignificant, and the main purpose of tranexamic acid is to decrease blood loss and not to decrease or change the risk of postoperative venous thromboembolism in musculoskeletal cancer patients [21]. Thromboprophylaxis is a new approach used to prevent the occurrence of postoperative venous thromboembolism, in which a pharmacologic agent is administrated in order to decrease the risk of thrombus formation. Mendez et al. found that aspirin decreases the risk of postoperative venous thromboembolism formation in patients diagnosed with malignant soft tissue carcinoma, metastatic carcinoma, lymphoma, or multiple myeloma when compared to another group that did not receive any thromboprophylaxis treatment [22]. The finding was found to be statistically significant. The study also identifies predicting factors that helps to determine which patients may benefit from thromboprophylaxis using aspirin, including patients with history of venous thromboembolism, history of diabetes melitus, operative blood loss around 250–1000 mL, and history of postoperative transfusion.

Another widely known agent that may be used as thromboprophylaxis is heparin, particularly low molecular weight heparins (LMWH). LMWH has been widely known as a pharmacological agent with beneficial role in preventing and treating thromboembolism. Medical guidelines have stated the utilization of LMWH injections to prevent postoperative thromboembolism in patients suffering from neoplasms with a risk of long-term immobilization [23]. Past clinical trials have found the efficacy of LMWH for cancer-associated thrombosis. LMWH is also recommended as a preventive and treatment agent for established venous thromboembolism and is used for at least 3 months [24, 25]. Even though LWMH may be considered as the best anticoagulant choice for patients with cancer, it also comes with downside. In some countries, LMWH is considered as an expensive drug, and the use of injection may lower compliance of some patients [26].

## 4. Conclusion

Malignant musculoskeletal tumour poses a threat to public health since it may cause significant morbidity and mortality. One of the most concerning complication associated with musculoskeletal tumour is the incidence of thromboembolism, which may happen as result of disturbance in the hemostatic equilibrium. Timely diagnosis using accurate diagnostic methods, while also considering significant predisposing factors together with right treatment, is beneficial in preventing disabilities caused by cancer-associated thromboembolism.

## Figures and Tables

**Table 1:** List literatures reviewed.

Studies	Year	Type of study	Findings of the study
*Pathophysiology*			
Falanga et al.	2012	Literature review	Risk of VTE in cancer patients is significantly higher compared to normal population
Walder et al.	2012	Case report	Invasion of tumour into vascular tissue and invasive treatment such as catheters may affect incidence
Falanga et al.	2015	Literature review	Musculoskeletal tumour inhibit fibrinolysis and cytokine production while increasing platelet aggregation
Hisada et al.	2015	Literature review	Central venous line, transfusion, and chemotherapy increase the risk of thrombus formation in cancer patients
Yoshimura et al.	2018	Prospective cohort	High BMI increases the risk of thrombus formation
Groot et al.	2018	Retrospective cohort	Risk of VTE increases significantly in patents with long bone metastasis receiving surgical therapy
Tukaye et al.	2016	Literature review	Thrombosis may be the first finding of occult tumour
Dickmann et al.	2013	Prospective cohort	Increased cumulative probability of VTE in cancer patients with metastases

*Diagnosis*			
Mitsunaga et al.	2013	Case report	Contrast-enhanced MRI may be helpful in finding tumour thrombus
Navalkele et al.	2013	Case report	The utilization of ultrasound in diagnosis of thromboembolism in cancer patients
Kaiser et al.	2017	Retrospective cohort	Preoperative complete blood examination may help in screening patients with risk of postoperative VTE

*Treatment*			
Haase et al.	2020	Retrospective cohort	Tranexamic acid may decrease the risk of postoperative VTE with insignificant result
Mendez et al.	2017	Retrospective cohort	Aspirin decreases the risk of thromboembolism in several types of cancer when administered as thromboprophylaxis
Farge et al.	2016	Guideline	LMWH has been widely used to prevent postoperative thromboembolism in patients with a high risk of immobilization
Watson et al.	2015	Guideline	LMWH may be used as a preventive and treatment agent for VTE
Kearon et al.	2016	Guideline	LMWH may be used as a preventive and treatment agent for VTE
Farge et al.	2019	Literature review	The limitation of LMWH is that it is an expensive drug and is administered by injection, therefore lowering compliance

VTE: venous thromboembolism; LWMH: low molecular weight heparin.

## Data Availability

The data used to support the findings of this study are available upon on request.
